# Early neurovascular retinal changes detected by swept-source OCT in type 2 diabetes and association with diabetic kidney disease

**DOI:** 10.1186/s40942-021-00347-z

**Published:** 2021-12-05

**Authors:** Monica Oliveira da Silva, Anne Elise Cruz do Carmo Chaves, Glauber Corrêa Gobbato, Mateus Augusto dos Reis, Fabio Lavinsky, Beatriz D’Agord Schaan, Daniel Lavinsky

**Affiliations:** 1 Department of Ophthalmology, Hospital de Clinicas de Porto Alegre, Federal University of Rio Grande do Sul, UFRGS, Porto Alegre, Brazil; 2 Retina and Vitreous Research Center, Hospital de Clinicas de Porto Alegre, Porto Alegre, Brazil; 3 Department of Endocrinology, Hospital de Clínicas de Porto Alegre, Porto Alegre, Brazil; 4 Graduate Program in Endocrinology, Federal University of Rio Grande do Sul, UFRGS, Porto Alegre, Brazil; 5 Medical School, UNISINOS University, Porto Alegre, Brazil; 6 Lutheran University of Brazil Medical School, Porto Alegre, Brazil

**Keywords:** Diabetes mellitus type 2, Glomerular filtration rate, Diabetic retinopathy, Optical coherence tomography, Retinal neurodegeneration

## Abstract

**Purpose:**

To evaluate retinal thickness and capillary density in patients with type 2 diabetes (T2D) and their association with diabetic kidney disease (DKD) using swept-source optical coherence tomography (SS-OCT).

**Methods:**

A cross-sectional study was conducted with T2D patients with mild or no diabetic retinopathy (DR) and nondiabetic controls. Inner retinal layer thickness was measured with SS-OCT. Retinal capillary density and the foveal avascular zone (FAZ) were measured with SS-OCT angiography (OCTA). SS-OCT parameters were compared in patients with and without diabetic kidney disease (DKD) and nondiabetic controls.

**Results:**

131 DKD eyes showed decreased ganglion cell layer plus (GCL+) (p = 0.005 TI; p = 0.022 I), retinal nerve fiber layer (RNFL) (p = 0.003), and central retinal thickness (CRT) (p = 0.032), as well as foveal avascular zone (FAZ) enlargement (p = 0.003) and lower capillary density in the superficial vascular plexus (p = 0.016, central quadrant), compared to controls. No statistically significant changes were found between diabetic patients without significant DKD and controls.

**Conclusion:**

Our findings suggest early neurovascular damage in patients with T2D; these changes were more significant in patients with DKD. Larger longitudinal studies are warranted to determine the role of early neurovascular damage in the pathophysiology of severe DR.

## Introduction

Diabetic retinopathy (DR) is the leading cause of vision loss and the most important ocular complication of diabetes mellitus [1]. Major risk factors are duration of diabetes, chronic hyperglycemia, and hypertension [2]. Chronic hyperglycemia leads to increased oxidative stress, inflammation, and hypoxia, all of which induce changes to the retinal neurovascular unit [3]. Diabetic retinopathy (DR) is classified clinically into a severity scale based on the presence of microaneurysms, hemorrhages, and vascular changes, such as venous beading, intraretinal microvascular abnormalities (IRMA), or neovascularization [4]. More advanced imaging technologies, such as optical coherence tomography (OCT), currently do not contribute to this classification, but they can detect early changes in retinal and vascular morphology in patients without DR or with mild DR [5].

Optical coherence tomography has become an important tool for management and follow-up of retinal diseases such as diabetic macular edema. Although most studies use the spectral domain OCT (SD-OCT), the recently introduced swept-source OCT (SS-OCT) has improved image penetration using a wavelength of 1050 nm, higher imaging speeds (axial scan rate of 100,000 scans per second), higher detection efficiency, improved imaging range, and improved depth with reduced sensitivity roll-off that is capable of generating images of both the vitreous and choroid simultaneously [6]. SS-OCT is also capable of performing OCT angiography (OCTA), that uses the motion contrast generated by flowing erythrocytes to enable a noninvasive, dye-free, depth-resolved visualization of the retinal vasculature levels with high speed and eye tracking, which significantly decreases motion artifacts [7].

The role of retina neurodegeneration in the pathogenesis of microangiopathy remains unclear but recent studies pointed that diabetic neurodegenerative abnormalities could play an important role in the pathogenesis of early stages of DR in a large proportion patients T2D [8]. Lynch and Abràmoff reported functional and structural aspects of DR that precede clinical changes, such as neural apoptosis of ganglion, amacrine, and Müller cells, increased expression of glial fibrillary acidic protein (GFAP) in Müller cells, inflammatory glial activation, and increased expression of neurotrophic factors, such as basic fibroblast growth factor and ciliary neurotrophic factor [9].

Neuroretinal alterations in patients with diabetes without DR or with early-stage DR have been detected by OCT, and include decreased retinal nerve fiber layer (RNFL), ganglion cell layer (GCL), and inner plexiform layer (IPL) thickness [10]. Recent studies using SD-OCT and OCTA demonstrated that diabetic eyes could exhibit retinal and choroidal vascular alterations, potentially including perifoveal capillarity loss and choroidal thinning with volume loss, even before the occurrence of visible signs of DR [11, 12].

The term neurovascular unit (NVU) was first applied to the blood–brain barrier and, subsequently, to the retina, to describe the intricate communication between neurons, glial cells, and vascular cells that together coordinate the retinal vascular flow with metabolic activity [13]. The microvascular complications of diabetes affect the eyes and kidneys, and are associated with different risk factors such as diabetes duration and blood pressure and lipid control [14]. DR and diabetic renal neurodegeneration (DRN) are reported to have a strong association, and similar molecular pathways appear to be involved in the development of diabetic kidney disease (DKD) and retinal microvascular injury [15].

Therefore, the aim of this study was to detect structural and vascular retinal changes in patient with type 2 diabetes (T2D) with or without kidney disease (DKD) using SS-OCT and OCTA. Our hypothesis was that T2D and DKD would be associated with early neurovascular damage.

## Methods

### Subjects

This cross-sectional study was conducted at a public teaching hospital in Southern Brazil between July 2018 and July 2019. The protocol was reviewed and approved by the Hospital de Clínicas de Porto Alegre Ethical Committee (registration number 20180186), adhering to the tenets of the Declaration of Helsinki, and written informed consent was obtained from all patients.

The inclusion criteria were confirmed T2D according to the standards of the American Diabetic Association (ADA) [16], age above 45 years, and capacity to consent. The exclusion criteria were history of any significant ocular disease, previous diagnosis of glaucoma, previous eye surgery, spherical equivalent outside ± 3 diopters, media opacities, clinical signs of moderate or severe DR or central macular edema, and previous bariatric surgery.

The control group was composed of health volunteers above 45 years old, who had recent (maximum 3 months) normal glycated hemoglobin (HbA1c) values (< 5.7%), no prior history of diabetes, and no prior or current history of kidney disease. Controls also could not present any ophthalmologic condition that could interfere on images evaluation as opacities and spherical equivalent outside ± 3 diopters.

In the T2D group, DKD was defined by the presence of a urinary albumin concentration (UAC) > 14 mg/L and/or reduced estimated glomerular filtration rate (eGFR; < 60 mL/min/1.73 m^2^) [17]. Estimated glomerular filtration rate was calculated using the CKD-EPI (Chronic Kidney Disease Epidemiology Collaboration) equation [18], 19. To reduces the effects of diurnal variations, all examinations were carried out in the morning. T2D patients were divided into two groups according to kidney function: mild or no DKD (nDKD) (eGFR ≥ 60 mL/min/1.73 m^2^ and/or UAC < 14 mg/L or higher) and DKD (eGFR < 60 mL/min/1.73 m^2^ and/or UAC ≥ 14 mg/L).

Both eyes of eligible T2D patients and controls were included in this study. All patients were subjected to a complete ophthalmologic examination that included slit-lamp biomicroscopy, indirect ophthalmoscopy, fundus photography, best corrected visual acuity (BCVA), SS-OCT, and OCTA. Age, gender, diabetes duration, HbA1c, and systemic hypertension were recorded. Diabetic retinopathy was graded by a masked experienced ophthalmologist as none, mild, moderate, severe nonproliferative, or proliferative, with or without macular edema, based on the International Clinical Diabetic Retinopathy classification system using fundus color images acquired with a Topcon DRI OCT Triton device [20].

### SS-OCT and OCTA Imaging

**Fig. 1:**
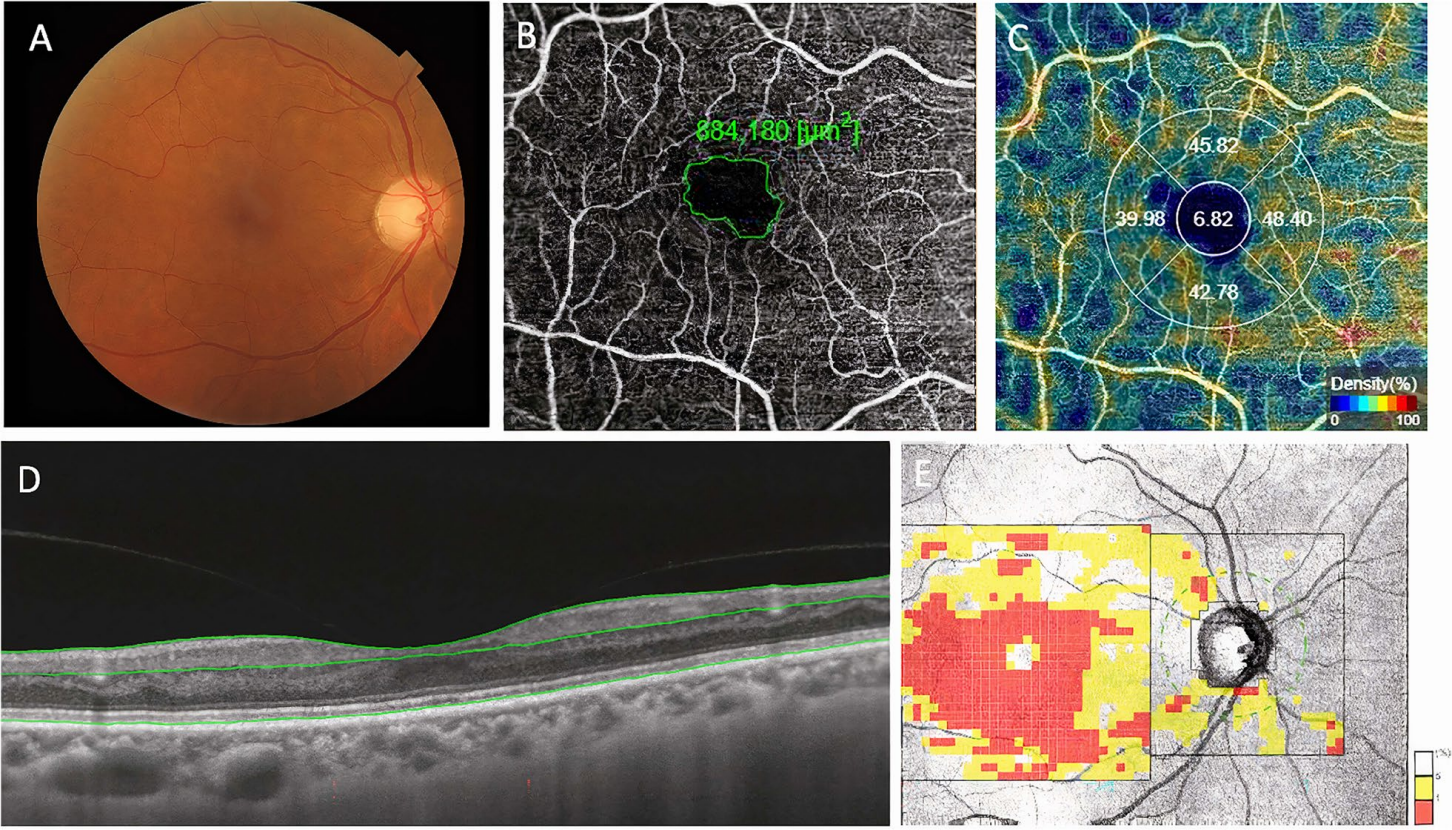
Representative case of a T2D patient with no signs of diabetic retinopathy (DR) on color fundus photography (**A**). SS-OCT with OCT-A shows enlargement of the foveal avascular zone (**B**), decreased capillary density (**C**) and thickness of inner retinal layers on structural OCT (**D**), and significant reduction of GCL + thickness (**E**)

SS-OCT and OCTA images were performed with a Triton DRI OCT plus scanner (Topcon, Tokyo, Japan), which uses a swept-source laser with a center wavelength of 1050 nm and scan speed of 100,000 A-scans per second. Images were analyzed using Triton Imaginet 6 (software version 1.22). In this study, the 3D(H) wide macula + line protocol was used with simultaneous color fundus picture of the posterior pole (Fig. 1). Before OCT imaging, pupils were dilated with tropicamide 0.5% at the same time of day to avoid diurnal variation.

All patients were evaluated through SS-OCT by an experienced ophthalmologist. OCT scans were excluded by the masked investigator if they showed signs of low quality or significant artifact. Choroidal/retinal thickness and vascular density measurements were obtained from an Early Treatment Diagnostic Retinopathy Study (ETDRS) grid centered at the fovea (diameters: center 1 mm, inner circle 3 mm, outer circle 6 mm). Automated segmentation of retina and choroid was performed in nine ETDRS areas plus average thickness, center thickness, and total volume. Ganglion cell layer plus (GCL+), from nerve fiber layer (NFL)/ganglion cell layer (GCL) to inner plexiform layer (IPL)/inner nuclear layer (INL) and GCL++ complex, from inner NFL interface to IPL/INL, and retinal nerve fiber layer (RNFL) segmentation was performed in six standard areas: superior (S), nasal superior (NS), temporal superior (TS), inferior (I), temporal inferior (TI), and nasal inferior (NI), plus total volume. An active eye tracker was used to reduce motion artifact.

OCTA scans were acquired using the DRI OCT Triton system based on the Topcon OCT angiography ratio analysis (OCTARA) algorithm. OCTA software uses the proportion of bright pixels to the proportion of dark pixels to derive a measure of vascular density. Capillary density values were obtained by applying a 6 mm × 6 mm ETDRS grid overlay centered at the fovea. Vascular density is derived from the percentage of the area occupied by bright pixels in a segmented area. Boundary layer segmentations were defined as superficial capillary plexus (SCP), from 2.6 μm below internal limiting membrane to 15.6 μm below the junction between IPL and INL (IPL/INL); deep capillary plexus (DCP), from 15.6 μm below the IPL/INL to 70.2 μm below the IPL/INL; choriocapillaris (CC), from Bruch’s membrane (BM) (0-μm offset) to 10.4 μm below the BM. Foveal avascular zone (FAZ) area (μm^2^) was measured using manual delineation in the superficial and deep plexus using native SS-OCT software.

### Statistics

Statistical analysis was performed using IBM SPSS software (version 26.0). Demographic and descriptive data were expressed as mean and standard deviation. For continuous variables, data were presented as the mean ± standard deviation; categorical variables were presented as percentages. Differences between groups were assessed with generalized estimating equations (GEE) and were adjusted by age and use of both eyes, excluding missing values.

## Results

Demographics and clinical characteristics of T2D patients and controls are summarized in Table 1. From 129 T2D patients, 258 eyes were included in the final analysis: 128 eyes of 64 subjects with mild or no DKD (nDKD), 130 eyes of 65 subjects with DKD, and 74 eyes of 37 controls. Diabetic patients were older, BCVA was lower in this group, and systemic hypertension was more prevalent than in controls. Among T2D subjects, we excluded 16 eyes due to moderate DR, 2 eyes with proliferative DR, 2 eyes with high opacity, 2 myopic eyes, 10 T1D eyes, and 2 MODY eyes.

**Table 1:** Demographic and clinical characteristics of T2D patients with no or mild diabetic retinopathy vs controls

Variable	T2D Group (129 subjects)	Control Group (37 subjects)	p-value
Gender, n (%)*			
Male	47 (36.4%)	13 (36.6%)	> 0.999
Female	82 (63.3%)	24 (63.4%)	
Age in years, mean (SD)	62.1 (± 8.85)	57.7 (± 6.4)	** < 0.001**
Ethnicity, n (%)*			
Caucasian	111 (85.9%)	30 (81.1%)	0.090
African descent/mixed	18 (14.1%)	7 (18.9%)	
BCVA, mean (SD)	45.2 (± 10.6)	52.7 (± 8.5)	** < 0.001**
Hypertension (%)	120 (93.2%)	13 (31.7%)	** < 0.001**
HbA1C, mean (SD)	8.3 (1.6)	5.5 (0.3)	** < 0.001**

Significant values in bold

BCVA, best corrected visual acuity

*Subjects

Table 2 summarizes characteristics between T2D subjects with no or mild DKD and DKD. This comparison showed no difference in HbA1c (8.2% ± 1.6 vs 8.4% ± 1.6, p = 0.513), hypertension (95.7% vs 91.5% p = 0.209), or age (61.3 ± 9.2 vs 62.9 ± 8.5, p = 0.144). Diabetic patients with DKD were more likely to have mild RD (72.9%, p < 0.001), and higher UAC (310.2 ± 878.6 mg/L, p ≤ 0.001). They presented also longer duration of DM (15.2 ± 7.3 years, p = 0.010), worse BCVA (43.7 ± 10.9 letters read, p = 0.022), and lower eGFR values (72.6 ± 27.7 mL/min per 1.73m^2^, p < 0.001).

**Table 2:** Demographic and clinical characteristics of patients with no or mild diabetic kidney disease (nDKD) and diabetic kidney disease (DKD) vs controls

Variable (n = 258 eyes of 129 study participants	nDKD (n = 128 eyes of 64 subjects)	DKD (n = 130 eyes of 65 subjects)	p-value
Gender, n (%)*			
Male	15 (23.4%)	32 (49.6%)	** < 0.001**
Female	49 (76.6%)	33 (50.4%)	
Age in years, mean (SD)	61.3 (± 9.2)	62.9 (± 8.5)	0.144
Ethnicity, n (%)*			
Caucasian	55 (85.9%)	58 (89.2%)	** < 0.001**
African descent/mixed	9 (14.0%)	7 (10.8%)	
Diabetic retinopathy stage, n (%)			
Without DR	114 (54.3%)	96 (45.7%)	** < 0.001**
Mild NPDR	13 (27.1%)	41 (72.9%)	
DM duration in years, mean (SD)	12.8 (± 6.4)	15.2 (± 7.3)	**0.010**
HbA1c, mean (SD)	8.2% (± 1.6)	8.4% (± 1.6)	0.513
BCVA, mean (SD)	47.1 (± 10)	43.7 (± 10.9)	**0.022**
Hypertension, n (%)	122 (95.7%)	119 (91.5%)	0.209
UAC (SD)	8.2 (± 5)	310.2 (± 878.6)	** < 0.001**
eGFR (SD)	89.3 (± 14.0)	72.6 (± 27.7)	** < 0.001**

Significant values in bold

BCVA, best corrected visual acuity (number of letters read); UAC, urinary albumin concentration; eGFR, estimated glomerular filtration rate

*Subjects

Retinal layers in patients with T2D were thinner than in controls in the following ETDRS locations (Table 3): inner retina, CRT, and total volume; GCL+ (all quadrants), GCL++ (all quadrants), and RNFL total and temporally.

**Table 3:** Mean retinal layer thickness in T2D and controls

Retinal Layer Thickness, ETDRS (μm)	T2D	Controls	95%CI	p-value
	(n = 258 eyes of 129 subjects) MD ± SD	(n = 74 eyes of 37 subjects) MD ± SD		
Retina				
Center Retinal Thickness (CRT)	237.8 (± 2.6)	252.6 (± 4.8)	− 24.5 to − 4.9	**0.003**
Total Volume	7.9 (± 0.04)	8.1 (± 0.1)	− 0.4 to − 0.1	**0.002**
Inner Temporal (IT)	293.1 (± 1.7)	307.9 (± 3.5)	− 17.3 to − 2.4	**0.010**
Inner Superior (IS)	309.9 (± 1.7)	321.6 (± 3.1)	− 18.6 to − 4.9	**0.001**
Inner Nasal (IN)	309.1 (± 1.9)	322.2 (± 3.0)	− 19.6 to − 6.5	**0.000**
Inner Inferior (II)	307.2 (± 1.7)	319.9 (± 2.7)	− 18.6 to − 6.8	**0.000**
GCL+				
Total	70.4 (± 0.6)	73.8 (± 1.0)	− 5.6 to − 1.2	**0.003**
Temporal Superior (TS)	70.5 (± 0.6)	74.0 (± 1.5)	− 6.6 to − 0.5	**0.024**
Superior (S)	69.7 (± 0.6)	73.3 (± 1.1)	− 5.9 to − 1.2	**0.003**
Nasal Superior (NS)	73.5 (± 0.6)	76.6 (± 1.0)	− 5.4 to − 0.9	**0.007**
Nasal Inferior (NI)	71.4 (± 0.7)	75.0 (± 1.0)	− 6.0 to − 1.2	**0.003**
Inferior (I)	66.6 (± 0.6)	69.6 (± 1.1)	− 5.4 to − 0.5	**0.017**
Temporal Inferior (TI)	71.0 (± 0.6)	74.46 (± 1.2)	− 6.0 to − 1.0	**0.007**
RNFL				
Total	105.5 (± 1.0)	110.0 (± 1.4)	− 7.9 to − 1.3	**0.007**
Temporal (T)	74.6 (± 1.0)	79.2 (± 1.5)	− 8.2 to − 1.0	**0.014**
Temporal Superior (TS)	136.6 (± 1.8)	146.1 (± 2.9)	− 16.3 to − 2.9	**0.005**
Nasal Superior (NS)	122.1 (± 1.9)	120.8 (± 4.0)	− 7.2 to 9.9	0.760
Nasal (N)	88.9 (± 1.2)	92.7 (± 2.2)	− 8.7 to 1.1	0.132
Nasal Inferior (NI)	137.1 (± 2.4)	140.0 (± 3.9)	− 12.0 to 6.2	0.534
Temporal Inferior (TI)	140.8 (± 1.9)	149.8 (± 2.8)	− 15.7 to − 2.3	**0.008**

Significant values in bold

GCL+, ganglion cell layer plus; RNFL, retinal nerve fiber layer

Mean capillary density values of superficial, deep plexus, and choriocapillaris are shown in Table 4. The superficial plexus of T2D patients presented lower capillary density compared to controls (central quadrant 95% CI, − 3.8 to 0.7, p = 0.004), except in the inferior quadrant. There were no vascular differences detectable in the deep and choriocapillaris layers (central quadrant 95% CI, − 2.5 to 1.2; p = 0.841 and 95% CI, − 2.5 to 0.4; p = 0.155).

**Table 4:** Mean OCT-A capillary density in T2D vs control groups

Vascular capillary plexus	T2D Group	Control Group	95%CI	p-value
	(n = 258 eyes of 129 subjects) MD ± SD	(n = 74 eyes of 37 subjects) MD ± SD		
Superficial				
Superficial Central (SC)	18.8 (± 0.4)	21.0 (± 0.6)	− 3.8 to − 0.7	**0.004**
Superficial superior (SS)	47.2 (± 0.3)	48.9 (± 0.5)	− 2.8 to − 0.5	**0.004**
Superficial Nasal (SN)	43.4 (± 0.3)	44.7 (± 0.5)	− 2.4 to − 0.1	**0.034**
Superficial Inferior (SI)	46.1 (± 0.3)	47.3 (± 0.6)	− 2.5 to 0.1	0.081
Superficial Temporal (ST)	45.5 (± 0.2)	46.4 (± 0.4)	− 1.8 to − 0.03	**0.041**
Deep				
Deep Central (DC)	18.0 (± 0.5)	18.6 (± 0.8)	− 2.5 to 1.2	0.481
Deep superficial (DS)	51.5 (± 0.3)	50.7 (± 0.5)	− 0.3 to 1.9	0.163
Deep Nasal (DN)	48.1 (± 0.2)	48.7 (± 0.5)	− 1.6 to 0.4	0.270
Deep Inferior (DI)	50.6 (± 0.3)	50.2 (± 0.6)	− 0.9 to 1.8	0.499
Deep Temporal (DT)	48.0 (± 0.2)	47.6 (± 0.4)	− 0.45 to 1.23	0.364
Choriocapillaris (CC)				
Central	48.7 (± 0.36)	49.8 (± 0.7)	− 2.5 to 0.4	0.155
Superficial	53.0 (± 0.1)	53.0 (± 0.3)	− 0.6 to 0.6	0.982
Nasal	52.8 (± 0.2)	53.4 (± 0.3)	− 1.3 to − 0.02	**0.042**
Inferior	53.0 (± 0.2)	53.4 (± 0.3)	− 1.1 to 0.3	0.270
Temporal	53.1 (± 0.2)	53.2 (± 0.2)	− 0.7 to 0.4	0.569
FAZ (foveal avascular zone)				
FAZ Superficial	313.8 (± 11.9)	243.4 (± 15.1)	31.1 to 108.8	** < 0.001**
FAZ Deep	188.5 (± 10.1)	137.1 (± 10.4)	21.8 to 79.1	**0.001**

Significant FAZ enlargement was observed in the diabetic group compared with control eyes in the superficial (95% CI, 31.1 to 108.8; p < 0.001) and deep plexus (95% CI, 21.8 to 79.1; p = 0.001) (Table 4). Analyzed separately, only DKD sample showed significant results: superficial plexus 95% CI, 19.2 to 116.5; p = 0.003 and deep plexus 95% CI, 17.1 to 96.9; p = 0.002 (Table 5).

**Table 5:** Statistical differences between nDKD and DKD subgroups vs control group

Retinal layers	Controls	nDKD (n = 128 eyes of 64 subjects)			DKD (n = 130 eyes of 65 subjects)		
	MD (µm) ± SD	MD (µm) ± SD	95% IC	p value	MD (µm) ± SD	95% IC	p value
Retina							
Center (CRT)	252.0 ± 4.4	239.1 ± 3.3	− 25.9 to 0.3	0.059	237.1 ± 3.3	− 28.7 to − 0.9	**0.032**
Total Volume	8.1 ± 0.1	7.8 ± 0.1	− 0.5 to − 0.1	**0.005**	7.9 ± 0.06	− 0.4 to − 0.02	**0.025**
Inner Temporal	308.3 ± 3.5	298.5 ± 2.3	− 19.7 to 0.3	0.061	297.5 ± 2.6	− 20.7 to − 0.7	**0.032**
Inner Superior	321.8 ± 3.1	309.6 ± 2.4	− 21.6 to − 2.7	**0.006**	310.1 ± 2.6	− 21.0 to − 2.3	**0.008**
Inner Nasal	322.2 ± 2.8	309.9 ± 2.5	− 21.3 to − 3.2	**0.003**	308.3 ± 3.0	− 23.1 to − 4.6	**0.001**
Inner Inferior	320.0 ± 2.6	307.8 ± 2.3	− 20.3 to − 4.1	**0.001**	306.6 ± 2.6	− 21.9 to − 4.9	**0.001**
GCL +							
Total	74.1 ± 1.0	70.7 ± 0.7	− 6.3 to − 0.5	**0.015**	70.0 ± 0.9	− 7.2 to − 0.9	**0.006**
Sup. Temporal	74.3 ± 1.5	70.5 ± 0.8	− 7.9 to 0.2	0.072	70.3 ± 0.9	− 8.2 to 0.2	0.065
Superior	73.6 ± 1.1	69.6 ± 0.7	− 7.1 to − 0.8	**0.008**	69.6 ± 0.8	− 7.3 to − 0.6	**0.014**
Sup. Nasal	76.9 ± 1.0	73.4 ± 0.8	− 6.4 to 1.0	**0.019**	73.3 ± 0.9	− 6.8 to − 0.3	**0.024**
Inf. Nasal	75.3 ± 1.0	71.8 ± 0.8	− 6.5 to − 0.4	**0.018**	70.8 ± 1.0	− 8.0 to − 1.0	**0.006**
Inferior	69.9 ± 1.1	67.1 ± 0.7	− 5.8 to 0.3	0.096	66.0 ± 1.0	− 7.3to − 0.4	**0.022**
Inf. Temporal	74.8 ± 1.1	71.8 ± 0.7	− 6.2 to 0.31	0.090	70.0 ± 1.0	− 8.4 to − 1.1	**0.005**
RNFL							
Total	110.5 ± 1.3	106.8 ± 1.3	− 8.2 to 0.7	0.131	103.9 ± 1.5	− 11.3 to − 1.8	**0.003**
Temporal	79.5 ± 1.5	76.0 ± 1.3	− 8.2 to 1.1	0.211	73.0 ± 1.4	− 11.7 to − 1.3	**0.009**
Sup. Temporal	146.8 ± 2.8	139.6 ± 2.4	− 15.9 to 1.5	0.148	133.3 ± 2.7	− 22.8 to − 4.1	**0.002**
Inf. Temporal	150.2 ± 2.7	144.2 ± 2.4	− 14.8 to 2.6	0.284	137.4 ± 2.7	− 22.3 to − 3.3	**0.004**
Sup. Nasal	121.1 ± 3.9	120.7 ± 2.8	− 11.9 to 11.0	1.000	123.3 ± 2.5	− 8.8 to 13.2	1.000
Nasal	93.1 ± 2.2	90.5 ± 1.7	− 11.7 to 7.8	1.000	87.1 ± 1.5	− 12.4 to 0.4	0.075
Inf. Nasal	140.9 ± 3.8	136.7 ± 3.4	− 16.1 to 7.7	1.000	136.9 ± 3.3	− 16.3 to 8.3	1.000
FAZ							
Superficial	241.8 ± 15.8	312.2 ± 17.7	− 117.8 to − 23.4	**0.004**	313 ± 14.5	− 114.5 to − 29.3	**0.001**
Deep	133.0 ± 10.5	182.0 ± 13.5	− 83.4 to − 14.7	**0.005**	194.0 ± 10.5	− 95.6 to − 26.4	**0.001**

Internal retina layers: GCL+, ganglion cell layer plus; RNFL, retinal nerve fiber layer

Significant values in bold

T2D patients with DKD has presented difference compared to controls, namely thinning of retinal central and inner temporal ETDRS quadrant, GCL+ temporal inferior and inferior quadrants, GCL++ temporal inferior quadrant, RNFL total, temporal, temporal superior, and temporal inferior quadrants. DKD and nDKD groups have presented independent differences compared to controls as registered on Table 5. However, we didn’t find any statistically significant difference between only nDKD group and controls.

**Fig. 2:**
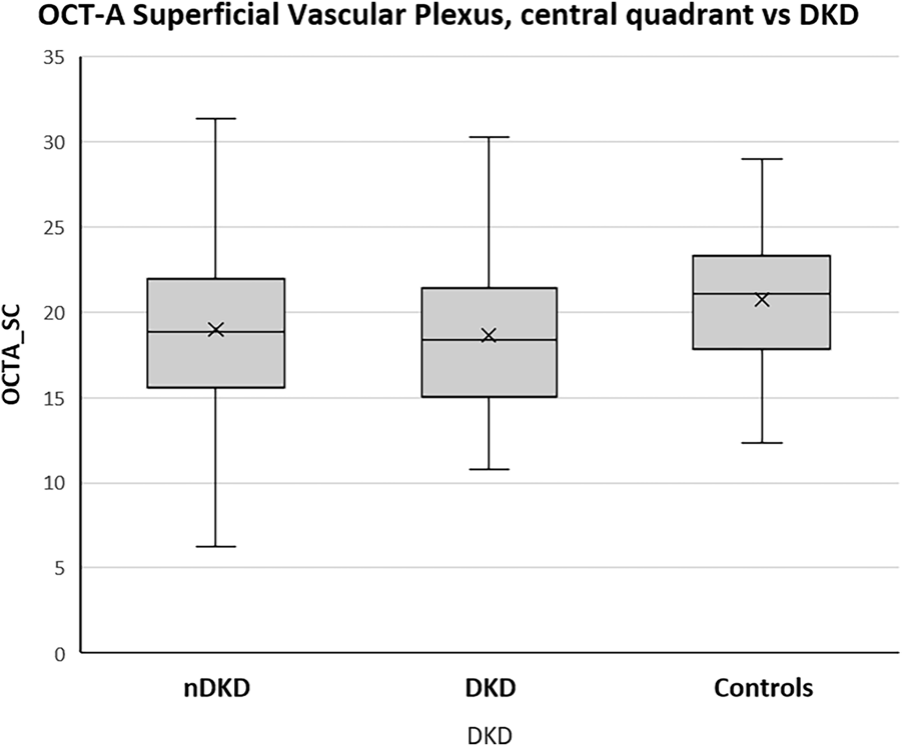
Comparative capillary density (%) of retinal superficial plexus between T2D nDKD and DKD subgroups and controls. The DKD subgroup showed significant loss of capillary density in the superficial central quadrant compared to controls

OCTA showed a lower capillary density in superficial plexus, central quadrant (95% CI − 4.0 to − 0.3; p = 0.016) in the DKD group compared to controls (95% CI − 4.2 to − 0.3; p = 0.020) (Fig. 2). No changes were observed in any groups regarding the deep vascular plexus (p = 0.762) and choriocapillaris (p = 0.208).

## Discussion

The inner retinal layers can be those most affected by diabetes, with reduction of thickness before clinically detectable vascular damage or in the early stages of DR. Most of this early damage can be detected using SD-OCT and enhanced depth imaging (EDI) mode. In our study, we used SS-OCT and OCTA to analyze retinal thickness and macular capillary density and the association of these findings with DKD in T2D patients and controls. Our results showed significant thinning of the inner retina of T2D patients, especially the GCL+/++ layer and RNFL; enlargement of the FAZ (both superficial and deep plexus); and lower capillarity in the superficial retinal plexus compared with controls. These findings suggest that neurovascular changes are an ongoing component of DR that may precede clinically moderate to severe microvascular changes.

The inner blood–retina barrier (iBRB) comprises the capillary endothelial cells which regulate blood flow in response to the metabolic demands of the retina, surrounded by pericytes, astrocytes and microglia; this complex is the neurovascular unit (NVU) [21]. iBRB integrity depends on the correct communication of the NVU component. Previous studies suggested that the retina in diabetes is subjected to successful adaptation to abnormal systemic conditions, followed by eventual loss of NVU function in response to progressive metabolic disruption, resulting in subtle clinical findings that could progress to advanced, vision-threatening retinopathy [22, 23].

Our study population with T2D had a mean disease duration of 13.2 ± 6.0 years in the nDKD group and 15.0 ± 8.0 years in the DKD group. Mean levels of HbA1c were 8.4% ± 1.8 in subjects without DKD and 8.3% ± 1.5 in the DKD group, which represents a long period of hyperglycemia that appears to be sufficient to induce disruption of NVU elements, but more severe DR elements such as IRMA, venous beading, and neovascularization were absent. However, the T2D group showed a significantly worse BCVA compared to controls, which could reflect functional consequences of this early damage to the neuroretina. Bao et al. described visual field defects in patients with diabetes and no clinically detectable DR, and suggested that early neuroretinopathy could precede the classical microvascular disease [24].

Along with these neurodegenerative findings, OCTA images showed a decrease of capillary density in the superficial plexus of the retina and an enlargement of the FAZ in patients with T2D. Reduction of capillary density and FAZ disruption are findings related to reduction of blood flow, caused by microvascular damage diabetes These findings corroborate previous descriptions of microcirculatory impairment, both in retinal and choroidal vasculature of diabetic eyes, before more severe findings of DR [25, 26], and add information about a possible association between signs of DKD and microvascular foveal changes that could indicate a higher risk for more severe DR.

Our findings suggest that reduction of superficial blood flow may be affecting primarily the ganglion cells and their axons (RNFL), corroborating that the NVU is subjected to early changes due to chronic hyperglycemia and that OCTA could be used along with fundus imagining for screening and follow-up of DR. These results can corroborate the potential of OCTA as a clinical tool for earlier detection of DR [25, 27].

RNFL was significantly thinner only among T2D patients with some degree of DKD, not in T2D patients without DKD or controls. A recent meta-analysis suggested that the retinal microvasculature signs can be associated with kidney diseases, provide significant data on concurrent kidney disease status and predict future risk for kidney disease development and progression [28].

The causal relationship between DR and DKD has already been more explored and evaluated. Most research data came from studies involving diabetic patients with DR diagnosed in more advanced stages (moderate to proliferative) [29, 30].

The scarcity of data on structural alterations in the retina of T2D patients with early DR or no DR and its link with kidney damage can be attributed, at least in part, to the sensitivity of the diagnostic devices used to examine for presence of DR, such as color fundus photography or fundoscopy. SS-OCT has a higher sensitivity than previous methods and offers a more accurate analysis of the retinal layers, even compared to SD-OCT [6]. Early anatomical and neurodegenerative changes in the retina in the absence of DR, or in its initial stages, can offer a new step to evaluate prognosis in the diabetic eye and even correlate it with kidney damage. According to Lynch and Abràmoff (2017), several lines of evidence now indicate that early neurophysiological and neurodegenerative changes should be considered as targets for therapy to supplement existing treatments for DR [9].

Our study had potential limitations, including its cross-sectional design and limited sample (composed mainly of white women). Also, there are natural age-related changes and thinning of neuroretinal parameters [31], hence we minimized this effect by adjusting the statistical analysis by age.

We reported that patients with kidney microvascular damage (DKD group) showed more retinal neurovascular alterations compared to patients without kidney disease (nDKD). However, these results of a cross-sectional design do not allow us to establish a causal relationship between kidney disease and diabetic retinopathy and it remains unclear whether diabetic neurovascular changes are a predictive, modulating, or causative factor in DR.

Longitudinal studies are necessary to evaluate if presence of early retinal neurovascular changes is associated to an increased risk of kidney disease or if presence of diabetic kidney disease can drive a worse retinal prognosis or even both conditions run independently.

Longitudinal studies are also warranted to determine the role of these early neurovascular changes in predicting the development of severe non-proliferative and proliferative DR. These findings are potentially useful for screening, follow-up, and treatment of patients with T2D, and may positively impact their visual and systemic outcomes.

## Conclusion

In conclusion, this study used swept-source structural OCT and OCTA and detected early changes in retinal thickness in the GCL+, GCL++, and RNFL, as well as FAZ enlargement and decrease of superficial retinal capillary density, in T2D patients—most notably, those with DKD—compared to controls. DKD was associated with inner retinal and superficial plexus vascular changes in T2D patients with mild or no DR, suggesting an association of eye and early kidney changes.

## Data Availability

Patient data are registered in patient medical charts and OCT device. According to Brazilian legal rules, data only can be accessed through patient or patient’s legal representative permission.
